# Clinical, humanistic, and economic burden of severe haemophilia B in adults receiving factor IX prophylaxis: findings from the CHESS II real-world burden of illness study in Europe

**DOI:** 10.1186/s13023-021-02152-1

**Published:** 2021-12-20

**Authors:** Tom Burke, Sohaib Asghar, Jamie O’Hara, Margaret Chuang, Eileen K. Sawyer, Nanxin Li

**Affiliations:** 1 HCD Economics, Daresbury, UK; 2 Faculty of Health and Social Care, University of Chester, Chester, UK; 3 uniQure Inc, Lexington, MA USA

**Keywords:** Haemophilia B, Factor IX, Burden, Cost, Bleeds, Health-related quality of life, Patient-reported outcomes

## Abstract

**Background:**

Real-world studies of the burden of severe haemophilia B in the context of recent therapeutic advances such as extended half-life (EHL) factor IX (FIX) products are limited. We analysed data from the recent CHESS II study to better understand the clinical, humanistic, and economic burden of severe haemophilia B in Europe. Data from male adults with severe haemophilia B receiving prophylaxis were analysed from the retrospective cross-sectional CHESS II study conducted in Germany, France, Italy, Spain and the United Kingdom. Inhibitors were exclusionary. Patients and physicians completed questionnaires on bleeding, joint status, quality of life, and haemophilia-related direct and indirect costs (2019–2020). All outcomes were summarised using descriptive statistics.

**Results:**

A total of 75 CHESS II patients were eligible and included; 40 patients (53%) provided self-reported outcomes. Mean age was 36.2 years. Approximately half the patients were receiving EHL versus standard half-life (SHL) prophylaxis (44% vs 56%). Most patients reported mild or moderate chronic pain (76%) and had ≥ 2 bleeding events per year (70%), with a mean annualised bleed rate of 2.4. Mean annual total haemophilia-related direct medical cost per patient was €235,723, driven by FIX costs (€232,328 overall, n = 40; €186,528 for SHL, €290,620 for EHL). Mean annual indirect costs (€8,973) were driven by early retirement or work stoppage due to haemophilia. Mean quality of life (EQ-5D) score was 0.67.

**Conclusions:**

These data document a substantial, persistent real-world burden of severe haemophilia B in Europe. Unmet needs persist for these patients, their caregivers, and society.

## Background

Haemophilia B is less prevalent than haemophilia A, with estimates of 3.8 and 17.1 cases per 100,000 males, respectively [1]. As such, the scientific literature tends to focus much more on burden and outcomes of patients with haemophilia A alone, or haemophilia at large (A and B, comprised mostly of patients with haemophilia A). However, patients with haemophilia B endure an ongoing clinical burden with substantial impairment of daily activities and health-related quality of life (HRQoL) [2–4]. Patients with severe disease (factor IX level ≤ 1% IU/dL; FIX) experience frequent spontaneous and recurrent joint bleeds that, without appropriate treatment, lead to early arthritis, deformity, and other long-term complications [5–7]. The burden of haemophilia B remains substantial and persistent [8–11]. Improved outcomes also come with an inherent treatment burden, as treatments are administered 2–3 times per week for standard half-life (SHL) FIX or, more recently, every 1–2 weeks for extended half-life (EHL) FIX [12, 13].

Haemophilia B and its treatment also bear a notable economic burden on patients and society [14–17]. The original CHESS study (known as CHESS I, “Cost of Haemophilia in Europe: a Socioeconomic Survey”) reported significant costs associated with severe haemophilia and its treatment, including reduced employment and lost wages among patients and their caregivers in Europe [18]. Since the data collection period of CHESS I in 2014–2015, haemophilia management and treatments have continued to evolve with the introduction and adoption of EHL and non-factor products into clinical practice. The CHESS II study, conducted in 2019–2020, sought to describe the comprehensive burden of haemophilia in Europe following these advances, including capturing real-world use of EHL treatment and up-to-date haemophilia care management practices. The holistic burden of haemophilia B among patients with severe disease, in particular, has been underserved in the literature. As these patients are likely to receive prophylactic therapy, we focused this analysis on patients with severe haemophilia B receiving prophylaxis. The development of inhibitors is also a rightful area for attention in the literature, and in keeping with our objective to address less developed areas of research, historically, we focused on patients without inhibitors for this analysis.

Here, we analyzed data from the recent CHESS II study to provide updated real-world findings on management practices, disease burden, and unmet needs of adults with severe haemophilia B receiving FIX prophylaxis in Europe.

## Results

A total of 132 CHESS II participants had severe haemophilia B, of whom 75 (57%) were on continuous FIX prophylaxis and were included in the analysis. Patient-reported data were available from 40 (53%) participants. The mean age was 36.2 years. Slightly fewer than half of the patients were receiving EHL prophylaxis (n = 33, 44%; Table 1). Overall, most patients 76% were receiving primary prophylaxis; mean (SD) duration of secondary prophylaxis was 4.5 (3.87) years. There was broad geographic distribution of patients with the United Kingdom contributing the highest proportion (29%), followed by Italy (23%) and Spain (20%). Demographic characteristics were generally similar between patients receiving SHL or EHL prophylaxis.

**Table 1:** Baseline characteristics of patients with severe haemophilia B in CHESS II

	All patients (n = 75)	SHL (n = 42)	EHL (n = 33)
Age, mean (SD)	36.2 (15.97)	33.7 (14.26)	39.3 (17.66)
Weight, kg, mean (SD)	77.3 (11.07)	74.0 (11.58)	79.9 (10.06)
Country, n (%)			
UK	22 (29)	15 (36)	7 (21)
Italy	17 (23)	11 (26)	6 (18)
Spain	15 (20)	9 (21)	6 (18)
France	11 (15)	4 (10)	7 (21)
Germany	10 (13)	3 (7)	7 (21)
Medical history, n (%)			
Anxiety	9 (12)	5 (12)	4 (12)
Depression	5 (7)	4 (10)	1 (3)
HIV	1 (1)	0	1 (3)
Hepatitis B	4 (5)	3 (7)	1 (3)
Hepatitis C	9 (12)	4 (10)	5 (15)
FIX prophylaxis treatment, n (%)			
EHL	33 (44)	–	33 (100)
SHL	42 (56)	42 (100)	–
Primary FIX prophylaxis, n (%)	57 (76)	29 (69)	28 (85)
Secondary FIX prophylaxis, n (%)	18 (24)	13 (31)	5 (15)

EHL, extended half-life; FIX, Factor IX; HIV, human immunodeficiency virus; SD, standard deviation; SHL, short half-life

Proportions may not sum to 100% due to rounding

Most patients with severe haemophilia B on FIX prophylaxis had ≥ 2 bleeding events per year (70%) and the mean ABR was 2.4 (Table 2). Mean number of bleed-related hospitalisations overall was 0.67, and appeared lower among patients receiving EHL prophylaxis. Twenty-three percent of patients had ≥ 1 target joint and 40% had ≥ 1 problem joint. ABR was similar between patients receiving EHL or SHL prophylaxis. History of joint surgery was similar between patients receiving EHL prophylaxis (33%) or SHL (24%), likewise for problem joints, but a greater proportion of patients receiving EHL reported target joints (Table 2).

**Table 2:** Clinical outcomes from patients with severe haemophilia B on FIX prophylaxis in CHESS II (past 12 months)

Clinical outcomes	SHL patients (n = 42)	EHL patients (n = 33)	All patients (n = 75)
ABR, mean (SD)^†^	2.4 (1.67)	2.5 (2.62)	2.4 (2.14)
Number of bleeds per year, n (%)			
None	6 (15)	5 (15)	11 (15)
1	6 (15)	5 (15)	11 (15)
≥ 2	28 (70)	23 (70)	51 (70)
History of joint surgery, n (%)^‡^	10 (24)	11 (33)	21 (28)
Bleed-related hospitalisations, mean (SD)^#^	0.76 (0.95)	0.55 (1.30)	0.67 (1.10)
Bleed-related hospital days per patient, mean (SD)	1.2 (2.9)	1.9 (5.2)	1.5 (4.1)
Target joints, mean (SD)	0.3 (0.71)	0.4 (0.79)	0.4 (0.74)
Number of target joints, n (%)	35 (83)	23 (70)	58 (77)
None	3 (7)	8 (24)	11 (15)
1	4 (10)	2 (6)	6 (8)
≥ 2			
Problem joints, mean (SD)	0.6 (0.94)	0.9 (1.32)	0.7 (1.12)
Number of problem joints, n (%)			
None	26 (62)	19 (58)	45 (60)
1	8 (19)	6 (18)	14 (19)
≥ 2	8 (19)	8 (24)	16 (21)

EHL, extended half-life; SD, standard deviation; SHL, short half-life

^†^ABR data were incomplete/missing for 2 patients

^‡^Types of surgery included arthrocentesis, arthrodesis, arthroplasty, arthroscopy, or synovectomy

^#^Bleed-related hospitalisation data were incomplete or missing for 13 patients

### Humanistic outcomes

The majority of patients reported mild or moderate chronic pain (76%), 5% reported severe chronic pain, and 19% reported no chronic pain. Most patients (63%) reported an impact of haemophilia on their daily lives and 20% reported adapting their treatment regimen in anticipation of physical activity. The overall mean EQ-5D score was 0.67 (standard deviation [SD], 0.21; Table 3). Due to small sample sizes, data are not reported separately for the SHL and EHL subgroups.

**Table 3:** Humanistic outcomes from patients with severe haemophilia B utilising FIX prophylaxis in CHESS II

Humanistic outcome reported by physicians	All patients (n = 75)
Chronic pain, n (%)	
No pain	14 (19)
Mild	34 (45)
Moderate	23 (31)
Severe	4 (5)

Patient-reported humanistic outcomes	All patients (n = 40)
Haemophilia impact on daily life, n (%)	25 (63)
Adapt treatment/physical activity, n (%)	8 (20)
EQ-5D score, mean (SD)	0.67 (0.21)

SD, standard deviation

### Economic outcomes

**Fig. 1:**
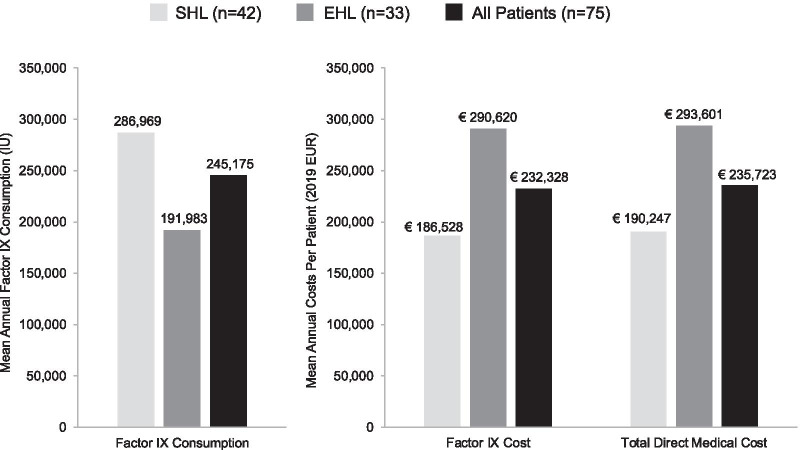
Mean annual FIX treatment consumption and direct medical costs. EHL, extended half-life; IU, international units; SHL, short half-life

**Fig. 2:**
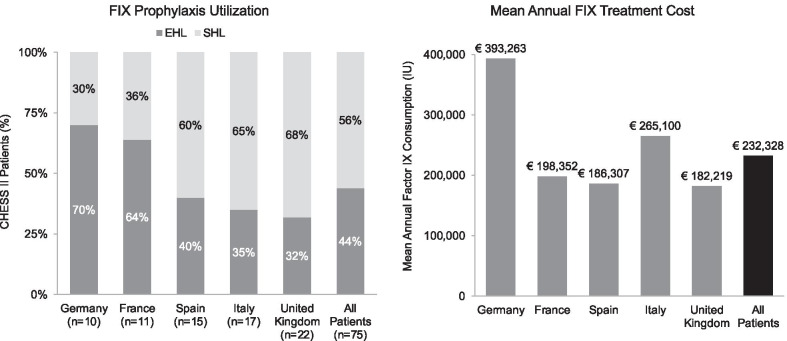
FIX treatment utilisation and costs by country. EHL, extended half-life; IU, international units; SHL, short half-life

The mean (SD) annual total haemophilia-related direct medical cost per patient was €235,723 (€154,953), comprised almost entirely of FIX treatment costs (€232,328; 99%). Total annual FIX consumption was 245,175 IU, and was higher among SHL than EHL patients (286,969 IU and 191,983 IU, respectively; Fig. 1). FIX treatment costs varied substantially by country, with the highest observed among patients in Germany (€393,263) and the lowest from those in the United Kingdom (€182,219; Fig. 2). Mean (SD) annual haemophilia-related non-medical direct costs were €1,997 (€3,187), driven by state entitlement payments (€1,082). Mean annual indirect costs were €8,973 (€15,398), driven by early retirement or work stoppage due to haemophilia (€5,389; Table 4).

**Table 4:** Mean annual indirect costs

	SHL patients (n = 25)	EHL patients (n = 15)	All patients (n = 40)
Total indirect costs	€11,907	€4083	€8973
Germany	–	–	€2447
Spain	–	–	€16,001
France	–	–	€36,809
Italy	–	–	€4340
UK	–	–	€3552
Early retirement/work stoppage	€7515	€1846	€5389
Germany	–	–	€2447
Spain	–	–	€16,001
France	–	–	€36,809
Italy	–	–	€4340
UK	–	–	€3552
Patient lost productivity	€1619	€1158	€1446
Germany	–	–	€2447
Spain	–	–	€426
France	–	–	€0
Italy	–	–	€654
UK	–	–	€3552
Caregiver lost productivity	€2773	€1079	€2138
Germany	–	–	€0
Spain	–	–	€6345
France	–	–	€0
Italy	–	–	€851
UK	–	–	€0

EHL, extended half-life; SHL, short half-life

Note: Sample sizes for country-level costs for each outcome were too small to be meaningful, and not included here

## Discussion

This analysis of the CHESS II real-world burden of illness study demonstrated the persistent negative impact of severe haemophilia B on patients in Europe. Patients averaged at least 2 bleeds per year, nearly half had at least 1 problem joint, and the majority reported mild or moderate chronic pain. Mean HRQoL was low for patients undergoing FIX prophylaxis, and indirect costs from early retirement/work stoppage and reduced work productivity for both patients and their caregivers were substantial. The direct costs associated with severe haemophilia B were substantial, driven almost entirely by FIX treatment.

This analysis of the recent CHESS II study provides a European perspective on real-world outcomes associated with FIX prophylaxis in patients with severe haemophilia B in the EU5. The study included a broad sample of patients receiving EHL or SHL treatment that was not limited to a single-center or a single-country analysis. These findings were even consistent with unmet needs reported prior to widespread use of EHL therapy for severe haemophilia B in Europe. Berntorp and colleagues reported real-world outcomes from haemophilia registries across 7 European countries where 57% of haemophilia B patients had severe disease (also 57% in CHESS II) [19]. Among patients with evaluable data for annual bleeds, Berntorp reported ≥ 1 bleed per year in 78% (57/73) of patients with severe haemophilia B (83% in CHESS II, 62/75) [19]. Other reports of the real-world clinical burden of severe haemophilia B in Europe included CHESS I, the predecessor of the current study [18, 20]. In this study, we observed a nearly identical distribution of ABR between patients receiving SHL or EHL treatment, but a greater proportion of EHL than SHL patients had at least one target joint (30% vs 17%, respectively). Physicians may have been more likely to prescribe EHL for their patients considered to have more severe joint complications, since switching to an EHL FIX product has been suggested to improve joint health outcomes [21], which has been discussed in the literature anecdotally but not quantified as of this writing [22, 23].

To our knowledge, CHESS II is the first multi-center study of EHL treatment use and costs among patients with severe haemophilia in the EU-5, specifically those with haemophilia B in this analysis. Though many patients had shifted to more expensive EHL therapy in CHESS II, the costs of haemophilia B in the EU5 were not substantially different from those observed with predominantly SHL therapy in CHESS I. Mean annual haemophilia-related direct medical costs for any FIX treatment were €232,000 in CHESS II, nearly identical to those reported with SHL treatment in CHESS I (€230,000) [18]. This may be due to changes in FIX unit cost associated with national tendering in EU, which varies with each country’s prioritization of prophylactic treatment in terms of both health policies and subsequent procurement. The proportional distribution of SHL and EHL treatment utilisation varied across countries in CHESS II, as did mean annual direct medical costs, which was likely due to differences in health system structures, policies, and reimbursement rates and was consistent with findings from CHESS I. The greatest proportion of EHL use and highest total costs were observed in Germany, which also showed the highest costs in the CHESS I analysis [18]. In general, we observed countries with lower proportions of EHL use to have lower total factor IX treatment costs, with the exception of Italy. This may have been due to health policies and/or procurement practices related to factor IX and EHL use specifically in this population.

Patients with severe haemophilia B in the CHESS II study showed substantial humanistic burden of disease and indirect costs despite FIX treatment. The majority of patients reported mild or moderate pain (76%) and impact of haemophilia on daily life (63%), consistent with published reports, particularly for patients with moderate to severe disease [4, 24]. The overall mean EQ-5D score among CHESS II patients with severe disease (mean 0.67) was slightly lower than that reported from severe haemophilia B patients in CHESS I (mean 0.76) [25]. This may be reflective of a persistent unmet need over time. We did not examine EQ-5D results by age due to the sample size of patients with severe disease; however, the mean overall EQ-5D score of 0.67 was substantially lower than published population norms for the participating countries (ranging from 0.86–0.92) [26]. The indirect costs of severe disease were observed to be similarly meaningful, comprised largely of early work stoppage or retirement (60%) followed by reduced productivity for caregivers (24%). The cost of lost productivity for patients was likely the smallest component of total indirect costs because early withdrawal from the workforce was the largest component, where severe haemophilia B may be more likely to keep working-age patients from working at all (mean age, 36 years in this cohort), rather than resulting primarily in absenteeism and presenteeism. Compromised employment due to severe haemophilia has been well established. Cutter and colleagues (2017) reported a negative impact of mild to severe haemophilia B on employment in the US as high as 95% among patients and 84–89% among caregivers and partners [27]. Patients who had left the workforce prematurely cited financial issues (59%), including healthcare expenses, and haemophilia-related issues (55%) most often.

Our analysis should be considered in the context of its strengths and limitations. The CHESS II study brings patient-reported outcomes and medical chart-extracted details to elucidate real-world encounters for patients with haemophilia B and is beholden to the expected limitations of any retrospective cross-sectional study, such as the potential for selection bias, recall bias, and data extraction errors. For example, patients who had sought physician care due to bleeding or joint health issues in the past 12 months were more likely to be included in the sample. The small sample sizes of patients with SHL or EHL prophylaxis precluded comparisons between these treatment groups, particularly for country-level and patient-reported outcomes. This analysis of the CHESS II data set focused on patients with severe haemophilia B without inhibitors with a record of consistent FIX prophylaxis over the past year. As such, our findings are not generalisable to patients with mild or moderate disease, those with inhibitors, and/or those receiving on-demand or less consistent prophylaxis therapy. Unmeasured variables may also impact the generalisability of our findings to other patient populations. Patient-reported outcomes (humanistic burden, indirect costs, and direct non-medical costs) were available from a subset of the overall study cohort (40/75 patients), which may have reflected a selection bias specific to those willing to complete the patient questionnaire. Our findings were from 5 large European countries, whereas recent studies of EHL costs across haemophilia types and subgroups have tended to be single-center, single-country, or more tightly focused regional analyses [28, 29].

## Conclusions

These findings from an analysis of the CHESS II study demonstrate the substantial clinical and humanistic burden of severe haemophilia B, together with a consistent economic burden. Our analysis provides a comprehensive assessment of real-world outcomes among patients receiving SHL and EHL FIX prophylaxis in Europe currently, highlighting the persistent unmet needs for these patients, their caregivers, and society.

## Methods

### Study design and patients

CHESS II is a retrospective burden of illness study of European adults with haemophilia that utilises a similar design and methodology as the original CHESS study, described elsewhere [18]. Briefly, CHESS II is a cross-sectional study of male adults (≥ 18 years old) with mild, moderate, or severe haemophilia A or B from eight European countries (Denmark, France, Germany, Italy, the Netherlands, Romania, Spain and the United Kingdom); however, sufficient data were only available from five countries (France, Germany, Italy, Spain and the United Kingdom) for this analysis. Patients and their haemophilia-treating physicians each completed questionnaires collecting information on self-reported health status, non-medical costs, and work impairment (from patients) and on the patient’s medical history (from the physicians) over the previous 12 months. Participating physicians recruited consecutive patients with haemophilia regardless of the reason for the clinical consultation visit. All data were collected between 2019 and 2020. The present analysis focused on patients with severe haemophilia B without a current inhibitor to FIX who received continuous EHL or SHL FIX prophylaxis in France, Germany, Italy, Spain and the United Kingdom.

The CHESS II study was approved by the Research Ethics Sub Committee of the Faculty of Health and Social care within the University of Chester and conducted in correspondence with regional and relevant guidelines. Patient consent for use of clinical data was not required as per European Pharmaceutical Market Research Association (EPhMRA) guidelines. Patient consent was obtained via tick box selection for the patient-reported element of the study.

### Study outcomes and analysis

In addition to demographic and clinical characteristics, we analyzed patient- and physician-reported clinical, humanistic, and economic outcomes from CHESS II. Clinical and economic outcomes were reported by the physicians based on review of the medical chart. A subset of patients reported humanistic outcomes including quality of life measures, with the exception of chronic pain which was reported by the physician. Demographic characteristics were collated from both patient- and physician-reported information. Clinical outcomes included frequency of bleeding events, annualised bleeding rate (ABR), bleed-related hospitalisations, target joints, problem joints, and joint surgery. The definition of a target joint was based on the International Society on Thrombosis and Haemostasis (ISTH) definition (“three or more spontaneous bleeds into a single joint within a consecutive 6-month period. Where there have been ≤ 2 bleeds into the joint within a consecutive 12-month period the joint is no longer considered a target joint”) [30]. A problem joint was defined as “any joint that has been permanently damaged as a result of a bleeding disorder, with or without persistent bleeding, and may involve chronic pain and/or limited range of movement due to compromised joint integrity such as chronic synovitis and/or hemophilic arthropathy” [31].

Humanistic outcomes included chronic pain, impact of haemophilia on daily life, adaptation of treatment regimen in anticipation of physical activity, and HRQoL based on responses to the EuroQoL 5-Dimension 5-Level (EQ-5D). Patients reported chronic pain over the previous 12 months on a scale of 0–10 (0 for “no pain,” 10 for “extreme pain”). Patient-reported work productivity impact was collected using the Work Productivity and Activity Impairment (WPAI) instrument (Reilly Associates, www.reillyassociates.net/wpai_general.html). Physicians reported FIX usage (IU) for each patient over the previous 12 months.

Economic outcomes included haemophilia-related direct medical costs, direct non-medical costs, and indirect and societal costs. Direct medical costs included medications (including over-the-counter medications), hospitalisations, physician consultations, professional caregiving assistance, medical devices, surgical interventions, and tests and procedures used for diagnosis and follow-up of haemophilia. Direct medical costs were calculated for each country, applying country-specific unit costs for each type of qualifying medical encounter extracted from the patient’s medical chart (Appendix Table 5). Direct non-medical costs included those for alternative therapies, home equipment and/or adaptations, transportation and transfer payments (including state benefits or disability allowances). Indirect costs included loss of wages and productivity due to absenteeism or impairment while at work both for patients and their informal caregivers, using the country-specific average salaries as a proxy for the opportunity costs. Components of each cost outcome and country-specific unit cost sources are provided in Appendix Table 6. Factor IX unit costs for each country are provided in Table 7.

**Table 5:** Country-specific direct medical unit cost sources

Country	Unit cost data source(s)
France	L’Assurance Maladie, https://www.ameli.fr Ministère des Solidarités et de la Santé, https://solidarites-sante.gouv.fr ViDAL, https://www.vidal.fr
Germany	Kassenärztliche Bundesvereinigung and Einheitlicher Bewertungsmaßstab, https://www.kbv.de/html/index.php Mein Pharmaversand, http://meinpharmaversand.de Rote-Liste Service, https://www.rote-liste.de
Italy	Ministero della Salute, https://www.trovanorme.salute.gov.it Tariffa minima degli onorari per le prestazioni medico-chirurgiche, https://www.ordinemedicilatina.it/files/521.pdf Starbene, https://www.starbene.it
Spain	Oblikue, http://esalud.oblikue.com Agencia Española de Medicamentos y Productos Sanitarios, https://www.aemps.gob.es
UK	National Schedule of Reference Costs, https://www.england.nhs.uk/national-cost-collection/ The Electronic Medicines Compendium, https://www.medicines.org.uk/emc#gref The NICE British National Formulary, https://www.nice.org.uk/bnf-uk-only

**Table 6:** Cost components used in the CHESS II study

Outcome	Component category	Measured element
Direct medical costs	Hospitalisations	Day case
		Outpatient (ie, for planned treatments)
		Inpatient, including length of stay
	Surgical procedures	Number and type of surgeries
		Length of stay
		Time spent in intensive care
	Consultant visits	Haematologist
		Other specialties
	Tests and examinations	Blood tests
		Other tests and examinations
	Coagulation factor	Brand (current and previous)
		Dosage
		Frequency
	Professional caregiver	Hourly wage
		Hours per week
Direct non-medical costs	Alternative and complimentary therapies	Number of visits
		Cost per session
	Travel costs	Car
		Public transport
	Requirement for aids/equipment	Walking aids
		Home adjustments
	Transfer payments	Entitlement per month
Indirect costs	Work productivity impact	Absenteeism
		Early retirement/stopped working
	Caregiver burden	Hours per week
		Work productivity impact for caregiver

**Table 7:** Factor IX unit costs (2021 unit costs unless noted otherwise)^a^

FIX unit cost (EUR)	UK	France	Germany	Italy	Spain
Alprolix®	–	0.96 (2018)^a^	1.47	1.38	1.24 (2020)^a^
Idelvion®	2.32	–	1.97	2.20	2.30 (2020)^a^
Refixia®	2.71	–	1.70	–	–
BeneFIX®	0.67^a^	0.65 (2011)^a^	0.87	0.73^a^	0.46
Rixubis®	0.67	0.65 (2017)^a^	0.77	0.69	0.46
AlphaNine®	0.43 (2018)^a^	–	0.72	0.41^a^	–

Unit costs were from the Pharmaceutical Pricing and Reimbursement Information (https://ppri.goeg.at/medicine_price_data)

^a^Other country-specific sources were as follows: for the United Kingdom, the British National Formulary (https://www.nice.org.uk/) and Branded Health Service Medicines Regulations 2018 (https://www.legislation.gov.uk/uksi/2018/345/contents/made); France, Modalités de prise en charge d’Aprolix (https://www.hematoalaune.fr/rubriques/vie-des-medicaments/modalites-de-prise-charge-dalprolix-eftrenonacog-alpha/#), Légifrance (https://www.legifrance.gouv.fr); Italy, Starbene (www.starbene.it); Spain, La Comisión Interministerial modifica el precio de 50 medicamentos (https://www.actasanitaria.com/la-comision-interministerial-modifica-el-precio-de-50-medicamentos/#:~:text=En%20cuanto%20al%20Alprolix%2C%20con,€%20para%20el%203000%20Ul)

All patient characteristics and outcomes were summarised using descriptive statistics, as means with standard deviations for continuous variables and as number and proportion of patients for categorical variables. All analyses were conducted using Stata 16.

## Data Availability

The data that support the findings of this study may be available from HCD Economics, Ltd but restrictions apply to the availability of these data, which were used under license for the current study, and so are not publicly available. Data may be available from the authors upon reasonable request and with permission of HCD Economics Ltd.

## Appendix

See Tables 5, 6 and 7.
